# A 14-bp insertion in endothelin receptor B-like (EDNRB2) is associated with white plumage in Chinese geese

**DOI:** 10.1186/s12864-020-6562-8

**Published:** 2020-02-17

**Authors:** Yang Xi, Lei Wang, Hehe Liu, Shengchao Ma, Yanying Li, Liang Li, Jiwen Wang, Han Chunchun, Lili Bai, Ahsan Mustafa, Hua He

**Affiliations:** 1 Farm Animal Genetic Resources Exploration and Innovation Key Laboratory of Sichuan Province, College of Animal Science and Technology, Sichuan Agricultural University, Chengdu, People’s Republic of China; 2 Institute of Animal Nutrition, Key Laboratory for Animal Disease-Resistance Nutrition of China, Ministry of Education, Sichuan Agricultural University, Chengdu, People’s Republic of China

**Keywords:** Geese, *EDNRB2*, Plumage color genetics, Genome and transcriptome sequencing, Fixation index analysis

## Abstract

**Background:**

Gang goose is a native species with gray plumage in Sichuan, China. As a result of overhunting, the number of gray Gang geese has decreased dramatically. To keep the species from extinction, conservation work for Gang geese was undertaken. In the process of pure breeding of gray Gang geese, approximately 2% of the offspring of each generation were white. This study aims to explain the genetic mechanism of this phenomenon and provide reliable molecular markers for goose-related plumage color breeding.

**Results:**

We used the method of pooled whole genome sequencing and *F*st (fixation statistics) to identify the differentiation degree of alleles between gray Gang geese and white Gang geese from their offspring. In this way, *EDNRB2*, a key gene that affects the migration of melanoblasts, was identified. Then, the transcriptome was sequenced for the two geese plumage color populations, and the DEGs (differentially expressed genes) were analyzed. The results indicated that *EDNRB2*, as a possible candidate gene, had a significantly differential mRNA expression. In addition, a 14-bp insertion (NW_013185915.1: g. 750,748–750,735 insertion. CACAGGTGAGCTCT) in exon 3 of EDNRB2 was analyzed and found to have a significant association between gray geese and Chinese white breeds (*P* = 0.00), while this mutation was not found in European geese. Meanwhile, the insertion was homozygous in all the white geese we detected and heterozygous in gray geese, indicating that this mutation is recessive. Furthermore, this 14-bp insertion leads to a frameshift mutation in the *EDNRB2* coding region and nonsense-mediated mRNA decay.

**Conclusion:**

Our study strongly suggests that the 14-bp insertion in exon 3 of the *EDNRB2* gene is associated with the white plumage phenotype in Chinese geese. This study is the first to investigate the relationship between *EDNRB2* and white plumage in geese.

## Background

Bird plumage color is a complex and diverse trait, with both color combinations and varied patterns, making birds the most colorful terrestrial vertebrates; therefore, these color variations have attracted interest from researchers. Plumage color plays an important role in attracting individuals of the opposite sex and avoiding predators [1, 2]. Moreover, in the poultry industry, plumage color is considered an important economic trait because the feather color directly shapes the consumer’s first impression and desire to purchase an ornamental bird. Feather color variants are mainly classified into basic colors, hues and patterns [3–5]. White feathers are generally classified into hue types.

White coat color is a common phenomenon for the various coat color types in livestock and poultry and is an important variant in animal breeding history. Functional defects in all stages of melanin synthesis may lead to a white coat color phenotype [6]. Many modern livestock and poultry breeds have white skin, hair or feather colors, including DLY (Duroc × Landrace × Yorkshire) pigs, AA (Arbor Acres), and Pekin ducks, and they are popular with consumers because of their white color and clean carcass appearance.

In chickens, three different genetic patterns of white feathers, including dominant white, recessive white and tyrosinase-independent recessive white, have been reported, and their dominant genes are *TYR* (tyrosinase), *PMEL17* (premelanosome protein) and *EDNRB2*. Scientists have found that a sequence of avian retroviruses was inserted into intron 4 of the *TYR* gene and that this insertion can lead to recessive white traits in chickens. This insertion results in the absence of exon 5 in transcriptional mRNA [7]. For the dominant white phenotype of chicken, sequence analysis showed that 9-bp insertions in exon 10 of the *PMEL17* gene were completely correlated with the allele of dominant white traits. This mutation also led to a 3-aa insertion in the transmembrane domain of the PEML17 protein [8]. Keiji et al. found a novel allele controlling the chicken white plumage mutation at the *mo* locus that harbors the coding gene *EDNRB2*; moreover, a Cys244Phe mutation was identified on *EDNRB2* in white plumage individuals of four Japanese chicken breeds [9]. In quails, the 2-bp deletion in exon 11 of the *MITF* (melanogenesis-associated transcription factor) gene can lead to a white plumage phenotype of quail homozygous state [10]. In ducks, the 6.6-kb insertion between exon 1 M and exon 2 of the *MITF* gene was confirmed to be the cause of duck white feathers. Moreover, this mutation has an epistatic effect on other color-related loci [11].

**Fig. 1:**
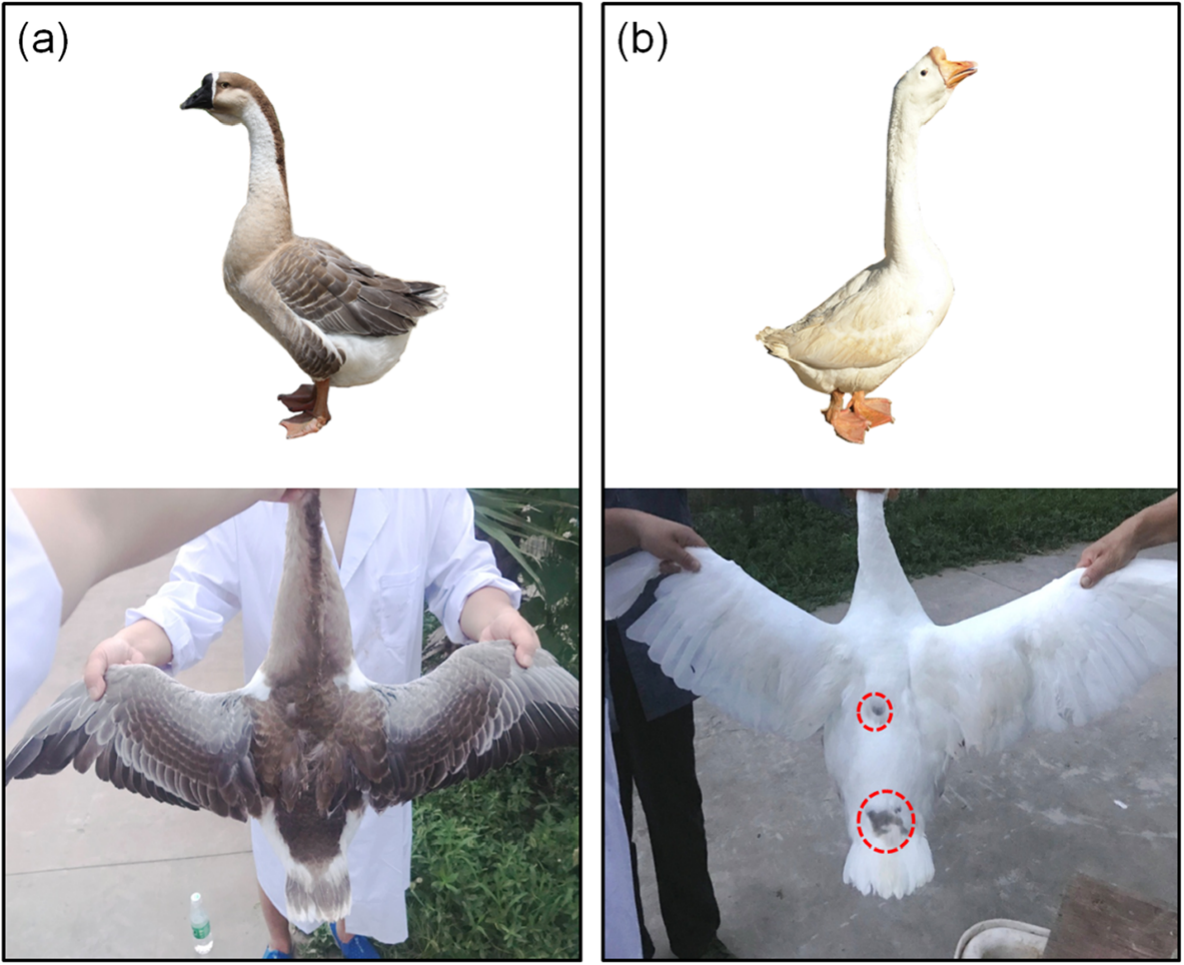
Phenotypic comparison between gray and white Gang geese. **a** A gray Gang goose. The feather color distribution of the gray Gang goose is more uniform. **b** A white Gang goose. Most of the feathers are white, but there are several black spots on the back (red circle marked). All the white geese in the breed conservation farm are descendants of gray Gang geese

Globally, geese are an economically important animal among other agricultural animals, and some of their breeds also exhibit white plumage phenomena. In China, most of the local goose varieties are white feathers, such as Zhedong white goose, Sichuan white goose, Wanxi white goose, and Zi goose. Gang goose is a local endangered gray feather variety present in Sichuan Province of China. According to statistics, in 2016, there were fewer than 2000 purebred Gang geese. Therefore, the breed conservation project for Gang geese was undertaken. However, in the process of pure breeding of gray Gang geese, approximately 2% of white geese will appear in each generation (Fig. 1). These white individuals have small pigmented spots on the dorsum, indicating that the mutation that causes the white plumage phenotype in Gang geese may not affect pigment synthesis while inhibiting the migration of pigment cells.

To date, there have been a number of studies examining geese plumage color and the underlying functional genes. Wang et al. suggested the effects of single nucleotide polymorphisms of candidate genes, i.e., *TYR* and *MITF*, and found a significant association between candidate genes and plumage color traits in domestic geese [12]. In the present study, genomic pooling resequencing and *F*st were used to identify a candidate gene and a possible causative mutation controlling the white plumage phenotype in Gang geese. Meanwhile, employing association analysis for different breeds, we found that the white feather mutation in Gang geese could be identified in many white goose breeds in China. This paper presents the first systematic study of geese plumage color and its associated genes.

## Results

### A genomic region on scaffold 394 is associated with plumage color

DNA samples were collected from a breed conservation farm. All the white Gang geese in this farm were segregated from the progeny of gray plumage geese. Twenty gray Gang geese and 20 white Gang geese were divided into two groups for genomic pooling sequencing.

The clean reads of pooled sequencing were mapped to the scaffolds on AnsCyg_PRJNA183603_v1.0 (NCBI accession GCA_000971095.1), which comprised 7593 scaffolds with a total size of 1,119,151,626 bp, and the mapping rates of both groups were greater than 90%.

**Fig. 2:**
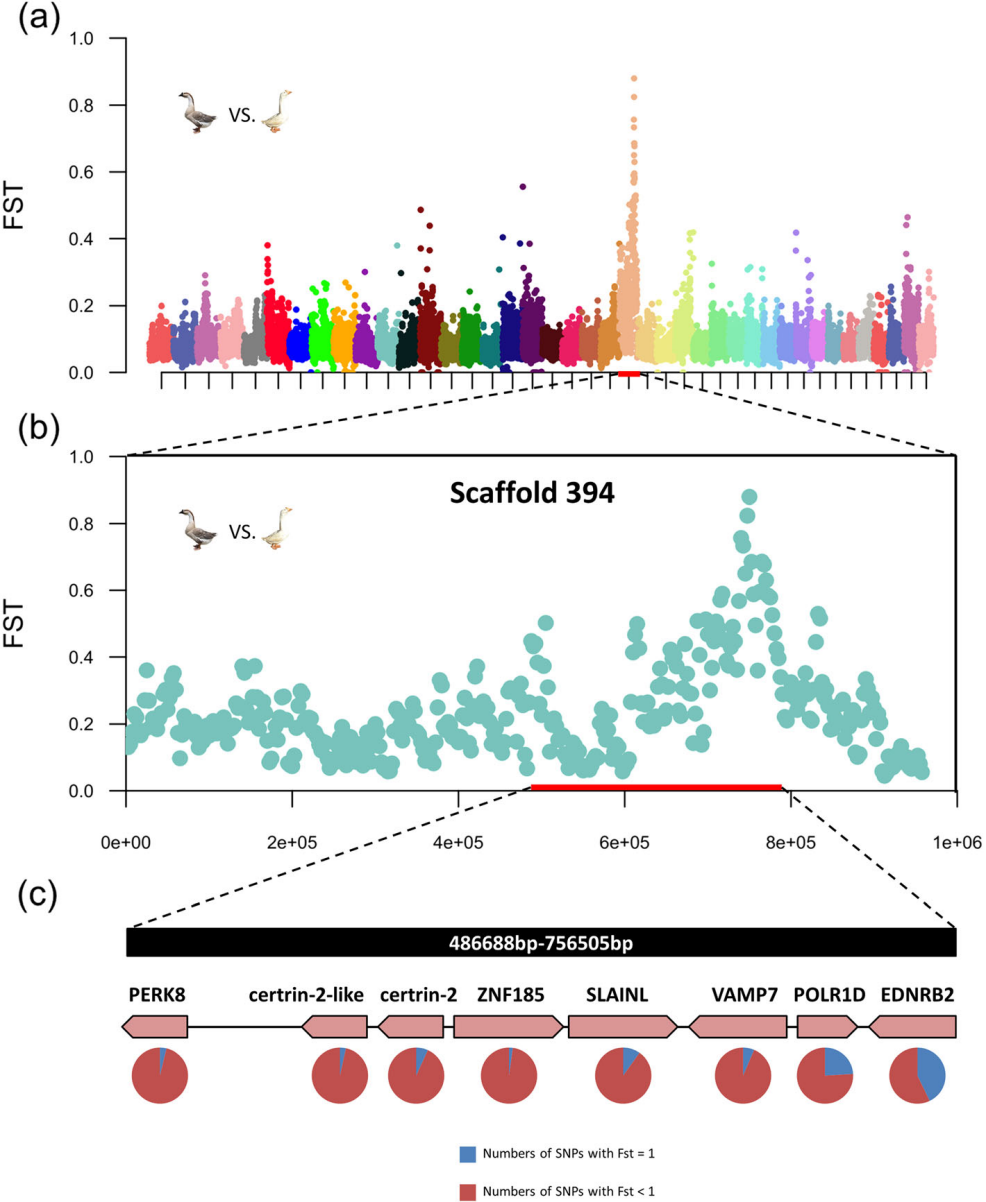
Manhattan plots for the white and gray plumage colors in Gang geese. **a** Whole-genome (scaffold 240–280) *F*st-value comparisons between the genomes of white and gray geese. The x-axis of the Manhattan plot shows the genomic position; the y-axis represents the *F*st values. All *F*st values were calculated in 5 kb windows. **b** *F*st of along scaffold 394 between white and gray geese. The red line represents the highest *F*st region. **c** Proportion of mutations with *F*st = 1 in each gene of the candidate genome region

To identify the genomic region containing the plumage color locus, *F*st was used to compare the resequenced genomes between gray Gang geese (*n* = 20) and white Gang geese (*n* = 20) (Figure S1). The *F*st value was calculated in 2.5 kb windows, and we selected windows with *F*st greater than 0.6 as potential candidate regions. Under such conditions, five windows were screened out, and 3 genes were included. Two of these 3 genes, LOC106047492 and LOC10607519, were in the 740,000-bp-752,500-bp range of scaffold NW_013185915.1, and another was in scaffold NW_013186074.1 (Table 1). The region with the highest *F*st value was on Scaffold NW_013185915.1 (Fig. 2a, b). In addition, four of the five previously filtered windows with *F*st values greater than 0.6 were all distributed within 1.25 kb of scaffold NW_013185915.1. Therefore, the proportion of mutations with *F*st = 1 of the 2 genes in scaffold NW_013185915.1 was calculated (Fig. 2c), and that in LOC106047519 was higher. We also performed synteny analysis of this region to confirm whether these two genes had annotations in the genome of other poultries. The results suggested that LOC106047492 and LOC106047519 were actually *POLR1D* and *EDNRB2*, respectively (Figure S2).

**Table 1:** Genes included in windows of *F*st > 0.6

Gene	scaffold	Pos	*F*st	Log_2_FC
*POLR1D* (LOC106047492)	NW_013185915.1	740,000	0.76	0.41
*EDNRB2* (LOC10607519)	NW_013185915.1	747,500	0.82	
	NW_013185915.1	750,000	0.88	1.21
	NW_013185915.1	752,500	0.69	
*LSD-like* (LOC106049694)	NW_013186074.1	15,000	0.77	0.01

Legend: The data in each column represent, from left to right, the gene names, the scaffold the genes are located in, the window position in the scaffolds, the *F*st values of each window and the Log_2_FC calculated by FPKM

### Transcriptome analysis indicated that *EDNRB2* was the candidate gene

To further reduce the scope of candidate genes, which were selected in the previous step, transcriptome sequencing was conducted from the feather follicles of 3 white Gang geese and gray Gang geese. The quality of clean data is demonstrated in the additional files (Table S1).

The genes were regarded as differentially expressed genes by the standard of |Log_2_FC| > 1, which are shown as volcano plots of differentially expressed genes (Figure S3). There were 416 differentially expressed genes between gray and white geese, among which 248 and 167 were up- and downregulated, respectively. Among the potential candidate genes screened out by *F*st analysis, only *EDNRB2* was differentially expressed (Table 1). Then, using the KEGG (Kyoto Encyclopedia of Genes and Genomes) database (www.kegg.gp), the pathways were enriched based on the differentially expressed genes using the criterion of *P* < 0.05 as significant. The mallard (*Anas platyrhynchos*) database was used as a reference species for KEGG analysis due to the slow update of the geese database. A total of 69 pathways were enriched, of which the top 1 was the melanogenesis pathway with 4 DEGs: *EDNRB2*, *TYR*, *TYRP1* (tyrosinase-related protein 1) and *MLPH* (melanophilin) (Figure S4).

### The 14-bp insertion in *EDNRB2* was associated with the white plumage of Chinese geese and decreased gene expression

**Fig. 3:**
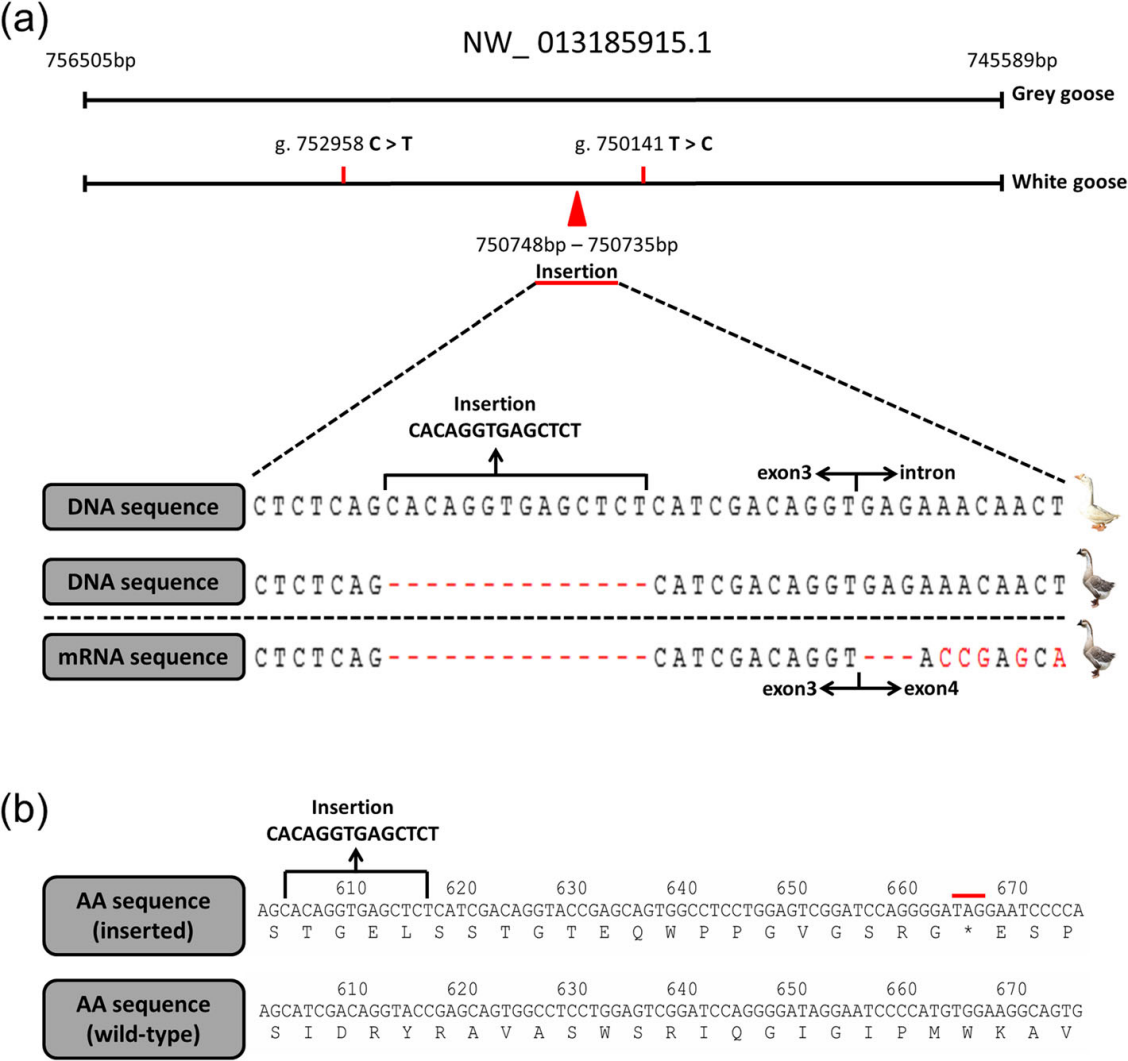
Sequence analysis of DNA, cDNA and amino acids of the insertion site. **a** The prediction of the boundary between exon 3 and intron and the position of the insertion sequence of white geese by DNA sequence alignment of the mRNA sequence of gray geese (the red arrow represents the position of the insertion sequence; the boundary of exon 3 and intron is indicated by a black arrow). **b** The prediction of the amino acid sequence of gray geese and white geese (the red line represents the potential pretermination codon)

The initial SNP (single nucleotide polymorphism) call provided 107 mutations in the *EDNRB2* gene sequence (NW_013185915.1: 745589 bp – 756,505 bp). Of these mutations, 35 SNPs and a 14-bp insertion were completely associated between the gray and white geese (Table S2); meanwhile, only 2 SNPs and the insertion were in the *EDNRB2* coding domain sequence (NW_013185915.1: g. 752,958 C > T, g. 750,141 T > C, g. 750,735–750,748 Insertion CACAGGTGAGCTCT; Fig. 3a). The 2 SNPs were synonymous mutations, but the 14-bp insertion in white geese led to a change in the amino acid sequence. We detected the insertion in 237 individuals from 8 geese breeds by analyzing chroma files and found that this mutation was significantly associated with the white plumage trait in Chinese geese, but the mutation does not exist in the white European breed (Table 2). The complete *EDNRB2* CDS (coding domain sequence) of gray geese was amplified, and we predicted the amino acid sequence (Figure S5 and Figure S6). The CDS sequence of gray Gang geese was compared with the DNA sequence of gray Gang geese and white Gang geese (Fig. 3b). The results indicated that the insertion was located on exon 3. Although we failed to amplify the cDNA sequence of the insertion site of white geese, the CDS region of *EDNRB2* carrying the 14-bp insertion was predicted for amino acid sequences, and a frameshift mutation was found; meanwhile, the pretermination codon appeared in the 664th to 666th bases of the CDS region (Fig. 3c).

**Table 2:** Association analysis of the 14-bp insertion in the white and gray Gang goose population

Breeds (Plumage color)	Genotype (Genotypic frequency)			*P*
	+/+	+/wt	wt/wt	
Gang goose (white)	32(1)	0(0)	0(0)	0.00^**^
Gang goose (grey)	0(0)	13(0.086)	138(0.914)	
Xingguo goose (grey)	0(0)	0(0)	3(1)	
Landaise goose (grey)	0(0)	0(0)	9(1)	
Zhedong white goose (white)	9(1)	0(0)	0(0)	
Wanxi white goose (white)	9(1)	0(0)	0(0)	
Sichuan white goose (white)	4(1)	0(0)	0(0)	
Zi goose (white)	10(1)	0(0)	0(0)	
Roman goose (white)	0(0)	0(0)	10(1)	

Legend: The genotype and genotypic frequency of each goose breed (+ for insertion; wt for wild-type). The chi-square test showed that there were significant differences between different plumage color populations (*P* < 0.01)

**Fig. 4:**
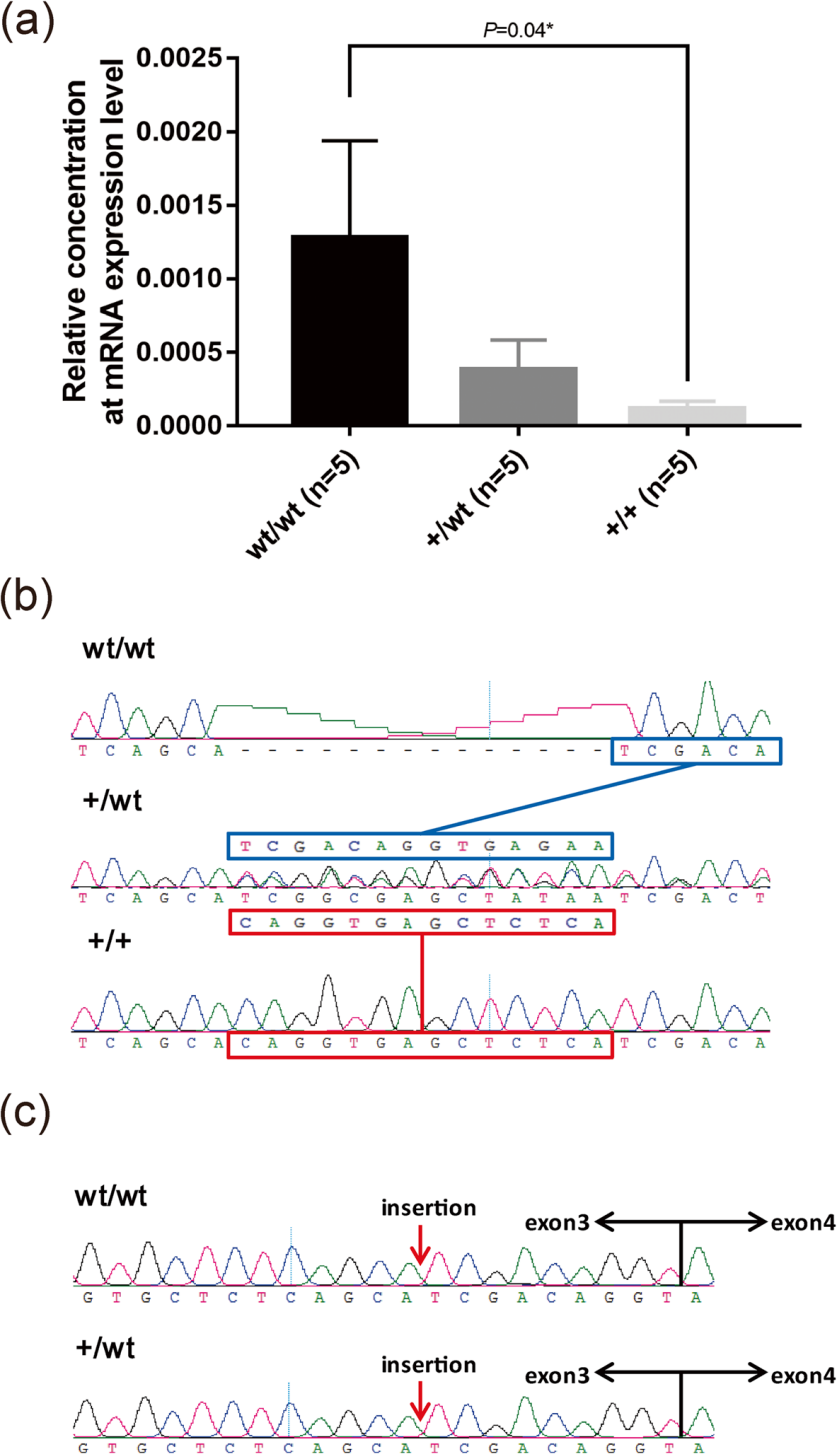
qRT-PCR and sequence analysis of heterozygous individuals. **a** Relative mRNA expression of EDNRB2 in individuals of three different genotypes. **b** Chromas files of heterozygotes at the 14-bp insertion site in the DNA sequence. The blue and red frames represent the DNA sequences in heterozygote individuals corresponding to the wt and insertion alleles, respectively. **c** The cDNA sequence of the insertion position of homozygous and heterozygous gray geese. The cDNA sequence of heterozygous individuals was the same as that of homozygous gray geese. No insertion was observed. The black arrow indicates the boundary between exon 3 and exon 4. The red arrow represents the location of the 14 bp insertion

This kind of faulty genetic message would lead to NMD (nonsense-mediated mRNA decay). To confirm this assumption, real-time quantitative PCR of *EDNRB2* in different genotypic individuals (wt/wt, wt/insertion, insertion/insertion) was conducted. Feather follicles were collected for RNA extraction from five individuals in each genotype group as biological repeats. The results indicated that the mRNA expression was the highest in individuals with the wt/wt genotype followed by those with the +/wt genotype, and there was little mRNA expression in the +/+ group (Fig. 4a). We also compared the sequence between genomic DNA and cDNA in homozygous and heterozygous gray geese. As expected, there was no insertion in the sequence at the mRNA level in heterozygous individuals (Fig. 4b, c).

## Discussion

In this study, a region in scaffold NW_013185915.1 was found to control the white plumage phenotype of Gang geese. By analyzing mRNA expression, the scope was further reduced, and *EDNRB2* was identified as the responsible gene. The association analysis of the mutations also provided strong evidence and indicated that a 14-bp insertion of *EDNRB2,* which could lead to the frameshift mutation, was significantly associated with other Chinese white goose breeds. In addition, through the results of association analysis, we can infer that the white plumage phenotype in Chinese geese should be recessive because we only observed the heterozygous genotype in the gray goose population. Meanwhile, in the pure breeding process of gray geese and white geese, only the offspring of gray goose had white individuals, but that of white geese had no character segregation. However, European geese did not carry a 14-bp insertion, indicating that this gene cannot explain the white plumage trait of European goose breeds. Similar phenomena can be explained in the study of chicken albinism [7–9]. Different genes in the melanogenesis pathway may lead to the same phenotype through different functions. The formation of feather color is mainly attributable to the deposition of melanin, and the deposition of melanin is determined by the development and migration of melanocytes [13, 14]. MITF is important for melanocyte proliferation, while TYR and TYRP1 play a role in melanin production in melanosomes [15–17]. Two of the most essential reasons why the hypopigmented phenotype appears were the decrease in the number of melanoblasts and abnormal cell migration [18–21]. *EDNRB2* clearly acts on the latter. The phenotypic variation caused by *EDNRB2* can be explained temporarily by studying its expression pattern and function. Compared with wild-type skin, the expression of *EDNRB2* in fibrous melanosis skin tissue was significantly increased in silky chickens [9]. The same result has been obtained in variant spotted quail, as well. The *EDNRB2* expression level in the skin of wild-type birds was significantly higher than that of mutant individuals [22]. In addition, scientists also found that the dorsal migration ability of melanoblasts was significantly reduced in *EDNRB2*-siSTRIKE-treated chicken embryos [23]. It is suggested that the expression level of *EDNRB2* plays an important role in melanin deposition on the body surface. From these studies, it can be concluded that the expression of *EDNRB2* is essential for melanin deposition, and it mainly inhibits the migration of melanoblasts to other places, resulting in a lack of pigment deposition in the body.

The 14-bp insertion on exon 3 of *EDNRB2* can explain why the mRNA expression level of *EDNRB2* decreased in heterozygous individuals. To date, coding region insertions similar to the one identified in present study have not been found in other birds or mammals. Through our prediction and analysis of the sequence, it could be inferred that the frameshift mutation might occur when the CDS region of white geese carried a 14-bp insertion. This kind of incorrect genetic information usually leads to RNA degradation, and this process is known as NMD (nonsense-mediated mRNA decay) [24]. Comparing the sequence of insertion sites of heterozygotes in genomic DNA and cDNA could provide strong evidence for this assumption. We could identify the two alleles, + and wt, in the genomic DNA sequence of the heterozygotes, but in the cDNA sequence, only the wt allele sequence was observed. Combined with our previous discussion on the expression and function of the *EDNRB2* gene, the most likely reason was that the insertion leads to the degradation of transcripts and reduces the expression in heterozygotes. Under this theory, our qPCR results could be explained, as well. The mRNA expression in heterozygotes was lower than that in homozygous gray geese. Moreover, the phenotypes of the 13 heterozygous individuals were recorded, and we found that there were several body parts without pigment deposition, such as the dorsum and primaries (Figure S7).

The above results indicate that the 14-bp insertion in *EDNRB2* plays an important role in controlling the white plumage phenotype of Chinese geese. This finding is helpful for understanding the transcriptome analysis performed in the present study. According to this analysis, in addition to *EDNRB2*, the mRNA expression of *TYR*, *TYRP1* and *MLPH* were also significantly different, and these genes were almost not expressed at the mRNA level in white feather follicles. In fact, after we confirmed that the responsible gene was *EDNRB2*, differential mRNA expression of any melanin synthesis-related genes became possible, since the impact of *EDNRB2* in pigment deposition is achieved by preventing melanoblast migration from the dorsal neural crest to body parts. As a result, there were probably almost no melanocytes in the feather follicles of white geese; therefore, it is reasonable that the expression of genes that play a role in melanocytes was low in the feather follicles of white individuals. This finding could also be the reason why we could not amplify the *EDNRB2* coding region of white goose in cDNA. However, according to the previous analysis, the expression of *EDNRB2* in any tissues of white geese should be very low because the two copies of the *EDNRB2* gene both carried the 14-bp insertion mutation, which could lead to NMD.

## Conclusions

Our study strongly suggests that a 14-bp insertion (NW_013185915.1: g. 750,735–750,748 insertion CACAGGTGAGCTCT) in *EDNRB2* exon 3 is associated with white plumage in Chinese geese. This study also proposed the mechanism governing the effect of this insertion on the geese white plumage phenotype. As the first study to explore the white plumage of geese, this study’s results help to characterize the white plumage of geese at the genetic level and identify molecular markers for geese breeding.

## Methods

### Animal

Twenty grey Gang geese and 20 white Gang geese were divided into two groups for genomic pooling sequencing. In addition, 3 of each grey Gang geese and white Gang geese were selected as transcriptome sequencing samples. Besides, the DNA was extracted from blood of 32 white Gang geese, 151 grey Gang geese, 9 Landaise geese (grey), 9 Zhedong white geese (white), 9 Wanxi white geese (white), 3 Xingguo geese (grey), 4 Sichuan white geese (white), 10 Zi geese (white) and 10 Roman geese (white, European white plumage breed), for insertion detection. Blood is collected from the veins under the wings. Samples of Gang geese were collected in National Gang Goose genetic Resources Conservation Farm. Other samples of Chinese local geese were provided by Shanghai Academy of Agricultural Sciences. No anesthesia was used in this study. We pull out one or two feathers of geese at the fastest speed, thus minimizing the stress of experimental animals. No goose individuals died in this study and all individuals stayed healthy after collecting blood and feather follicles.

All the experimental procedures, described below, were approved by the Animal Ethics Monitoring Committee of Sichuan Agriculture University, and carried out in accordance with Guideline of Animal Welfare China.

### Genome sequencing

The DNA samples from 20 grey Gang geese and 20 white Gang geese were extracted by using a standard Phenol-Chloroform extraction protocol. Afterwards, the concentration of each sample was diluted to 100 ng/μL according to the concentration of DNA mother liquid detected by Nanodrop 2000. All individual DNA samples, for each plumage colour, were pooled in a centrifuge tube, and then the pooled of DNA samples were used for the library construction and subsequent sequencing on platform of Illumina® Hiseq X-Ten platform to average raw read sequence coverage of 20X. DNA from each pool was fragmented by nebulization. After end repair and A-tailing, individual indexed PE (Paired end) adapters were ligated to the DNA fragments, which allowed multiplexed PE sequencing. The adapter-ligated fragments were size selected on a 2% low melt agarose gel to a size of 500–800 bp. After enrichment PCR of fragments that carry adapters on both ends the final libraries were quantified by PicoGreen. The average fragment size of each library was determined on a BioAnalyzer High Sensitivity DNA chip. Samples from each library were sequenced on a HiSeq2000 in rapid mode, as well as in high output mode. After completion of the sequencing runs, SNP calling, demultiplexing and FASTQ file generation were performed by using a CASAVA-based inhouse script.

### Mapping and SNP calling

The assembled goose genome AnsCyg_PRJNA183603_v1.0 (NCBI accession GCA_000971095.1, annotation release Apr 2015), which comprises 7593 scaffolds with a total size of 1,119,151,626 bp, was used as reference. Mapping of the Illumina sequencing reads onto the reference genome was conducted in the white and grey pools using the software BWA [25]. Local re-alignment and re-calibration were performed by using the GATK (Genome Analysis Toolkit, version 3.1) framework [26]. The initial SNP discovery was performed using multi-sample SNP-calling procedure in the GATK package. To reduce the false discovery rate, the filtering steps was conducted by using these criteria.

### *F*st analysis

The aligned reads were further calculated for *F*st by using Popoolation2 [27]. The *F*st values were averaged over SNPs in a 5 kb sliding window with a 2.5 kb step size for each comparison groups. Manhattan diagram of *F*st value is made by R-studio, using package ‘qqman’ [28].

### RNA extraction and Transcriptome

The calamus, in growing period, was used as the material to extract total RNA from geese feather follicle [29]. Calamus was put into lysing matrix (MP Biomedicals, LLC) containing 1 mL TRIzol reagent. The tubes were then ground for 30 s by tissue crushing apparatus (MP FastPrep-24™). Total RNA was extracted according to the manufacturer’s protocol. The quality and quantity of RNA samples were checked by Spectrophotometer NanoDrop 2000 and denaturing agarose gel electrophoresis. All RNA samples were treated with DNAse-I for later use.

Previously extracted RNA was used for RNA sequencing and library construction. The RNA-seq library preparation was performed following the protocol recommended by the manufacturer (Illumina, San Diego, CA, USA). In brief, after purification, the mRNA was fragmented into small pieces, and the first-strand cDNA was prepared by using random hexamers. Then the second strand cDNA synthesis, end repair, addition of a single A base, and ligation of the adapters were performed. The products were purified and enriched by PCR to create the final cDNA library. The mRNA sequencing was done on the Illumina NovaSeq with paired end 2 × 100 nt multiplex. The quality of clean data is evaluated by fastqc software.

### DEGs analysis

After the quality evaluation of the sequencing data, Hisat2 [30] software was used to compare the filtered clean reads to the goose AnsCyg_PRJNA183603_v1.0 (NCBI accession GCA_000971095.1, annotation release Apr 2015). The log_2_FC > 1 or < − 1 was used as a threshold to screen the differentially expressed genes. The enrichment analysis, of the differentially expressed genes, was conducted through KEGG (Kyoto Encyclopedia of Genes and Genomes) in KOBAS 3.0.

### Insertion detection

The SNPs and short INDELS (Insertion/Deletion) of *EDNRB2* were screened by variant calling. It was found that the nucleotide deletion of 14 bp, in the DNA, was found at the junction between the 3′ of exon 2 and the intron. To verify this result, 3 pairs of primers (Table S3) were designed to detect the insertion and amplify the whole CDS in white and grey geese. Amplification conditions were as follows: 3 min at 94 °C; 35 amplification cycles of 10s at 94 °C, 30s at annealing temperature, 120 s at 72 °C; 5 min at 72 °C. The sequence concluding the 14 bp-insertion in genomic DNA and cDNA was obtained by Sanger sequencing (Tsingke, China).

### qRT-PCR

Three pairs of primers were designed for detecting *EDNRB2* and the two reference genes (β-actin, GAPDH) by the Primer Premier 5.0 software. PCR reactions were carried out by using primers β-actin-F/R, GAPDH-F/R and *EDNRB2*-F/R (Table S3). RNA extraction from feather follicles was carried out in 5 random samples from each genotype group. TAKARA PrimeScript™ RT reagent kit was used in reverse transcription. Amplification conditions were as follows: 5 min at 94 °C; 35 amplification cycles of 20s at 94 °C, 20s at annealing temperature, 20s at 72 °C; 5 min at 72 °C. All reactions contained 3 replicates. Gene expression level was analysed using the 2^-∆∆CT^ method [31].

## Supplementary information

**Additional file 1: Figure S1.** Manhattan plots for the plumage colors Fst of white and gray in gang geese. **A.** Whole genome. **B.** Scaffold 1–23. **C.** Scaffold 24–76. **D.** Scaffold 77–156. **E.** Scaffold 157–231. **F.** Scaffold 232-end.

**Additional file 2: Figure S2.** The synteny blocks analysis of candidate region. Different colors represent different genes. Arrow direction is the direction of gene transcription.

**Additional file 3: Figure S3.** The Volcanic Plot of differentially expressed genes. A total of 416 differentially expressed genes in goose feather follicles, among which 248 and 167 were up- and down-regulated, respectively.

**Additional file 4: Figure S4.** KEGG annotations of differentially expressed genes. A total of 69 pathways enriched the differentially expressed genes.

**Additional file 5: Figure S5.** Complete sequence of grey goose CDS. A pair of primers was used to amplify the complete CDS sequence of gray goose, totaling 1359 bp.

**Additional file 6: Figure S6.** The predicted amino acid sequence of gray goose (left) and after insertion (right). The complete CDS of gray Gang goose (left) was amplified. The amino acid sequences of gray and white goose were predicted. In the gray goose, 452 amino acids were completely translated. However, the insertion (marked in green) may cause a frameshift mutation in the white goose. Pre-termination codon (marked in red) appears at the 664th to 666th nucleotides in the coding region.

**Additional file 7: Figure S7.** The phenotypic difference between the homozygote (wt/wt) and heterozygote (+/wt) in gray Gang geese. Figure A and C were the homozygote individuals while B and D were heterozygote. The red dotted circles represent the body areas with no pigment distribution. A. The dorsum of homozygote gray goose. B. The dorsum of heterozygote gray goose. C. The primaries of homozygote gray goose. D. The primaries of heterozygote gray goose.

**Additional file 8: Table S1.**

**Additional file 9: Table S2.**

**Additional file 10: Table S3.**

## Data Availability

The raw data of genome and transcriptome sequencing had been uploaded to SRA database. Accession ID: PRJNA556795.

## Abbreviations

AA: Arbor Acres
ANOVA: Analysis of variance
CDS: Coding domain sequence
DEG: Differential expression gene
DLY: Duroc × Landrace × Yorkshire
EDNRB2: Endothelin receptor B-like
*F*st: Fixation statistics
GATK: Genome Analysis Toolkit, version 3.1
INDEL: Insertion/Deletion
KEGG: Kyoto Encyclopedia of Genes and Genomes
LSD: Least-Significant Difference
MITF: Melanogenesis associated transcription factor
MLPH: Melanophilin
NMD: Nonsense-mediated mRNA decay
PE: Paired end
PMEL17: Premelanosome protein
SNP: Single Nucleotide Polymorphism
TYR: Tyrosinase
TYRP1: Tyrosinase-related protein 1
