# Supplementary material for: A 14-bp insertion in endothelin receptor B-like (EDNRB2) is associated with white plumage in Chinese geese
Source: BMC Genomics. 2020 Feb 17;21:162. doi: 10.1186/s12864-020-6562-8 (PMC7027040; doi:10.1186/s12864-020-6562-8)
Supplement: Supplementary file 1 — Additional file 1: Figure S1. Manhattan plots for the plumage colors Fst of white and gray in gang geese. A. Whole genome. B. Scaffold 1–23. C. Scaffold 24–76. D. Scaffold 77–156. E. Scaffold 157–231. F. Scaffold 232-end. [file 12864_2020_6562_MOESM1_ESM.docx]

**Figure S1. Manhattan plots for the plumage colors Fst of white and gray in gang geese. A.** Whole genome. **B.** Scaffold 1-23. **C.** Scaffold 24-76. **D.** Scaffold 77-156. **E.** Scaffold 157-231. **F.** Scaffold 232-end.
