# Supplementary material for: A 14-bp insertion in endothelin receptor B-like (EDNRB2) is associated with white plumage in Chinese geese
Source: BMC Genomics. 2020 Feb 17;21:162. doi: 10.1186/s12864-020-6562-8 (PMC7027040; doi:10.1186/s12864-020-6562-8)
Supplement: Supplementary file 2 — Additional file 2: Figure S2. The synteny blocks analysis of candidate region. Different colors represent different genes. Arrow direction is the direction of gene transcription. [file 12864_2020_6562_MOESM2_ESM.docx]

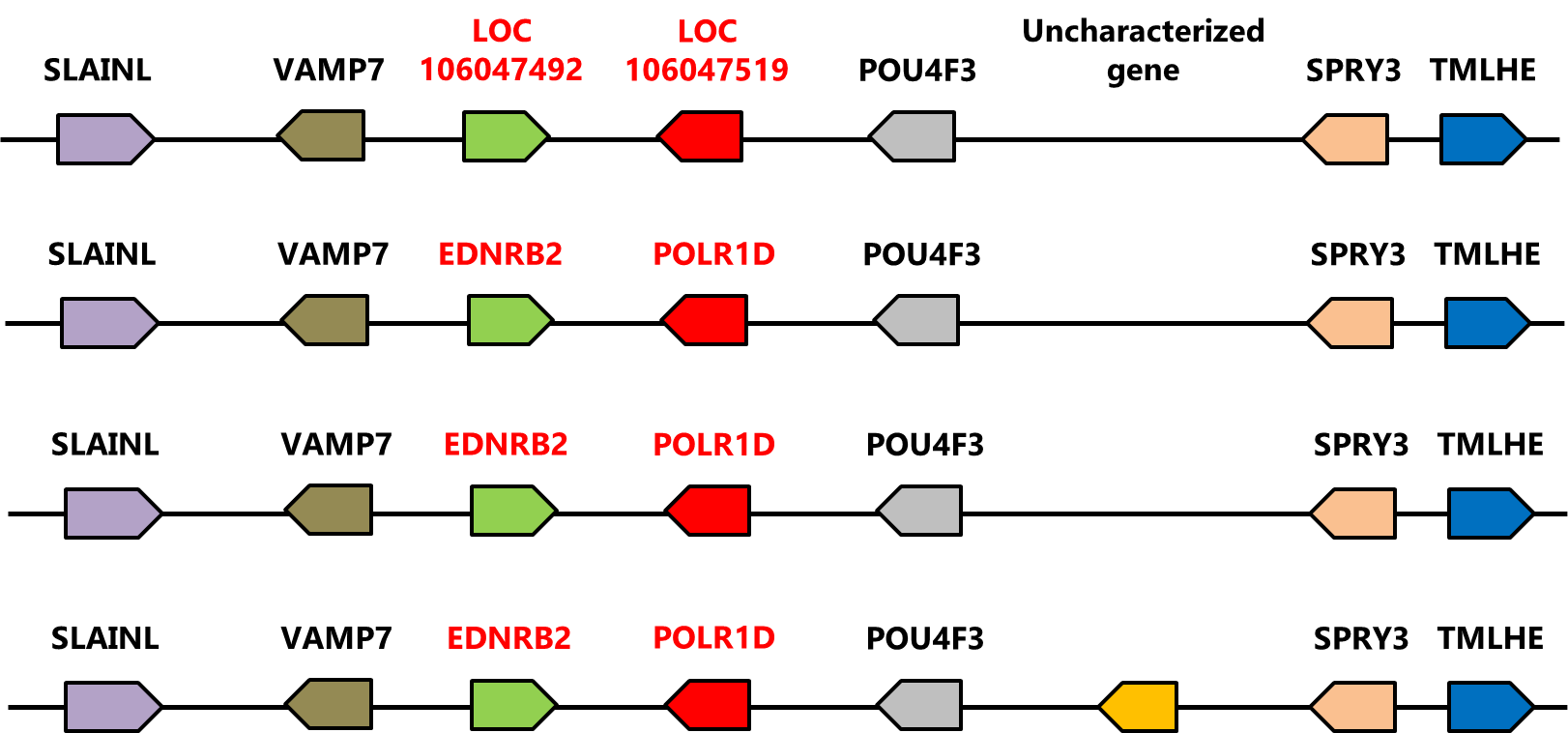


**Figure S****2.** **The synteny blocks analysis of candidate region.** Different colors represent different genes. Arrow direction is the direction of gene transcription.
