# Supplementary material for: A 14-bp insertion in endothelin receptor B-like (EDNRB2) is associated with white plumage in Chinese geese
Source: BMC Genomics. 2020 Feb 17;21:162. doi: 10.1186/s12864-020-6562-8 (PMC7027040; doi:10.1186/s12864-020-6562-8)
Supplement: Supplementary file 3 — Additional file 3: Figure S3. The Volcanic Plot of differentially expressed genes. A total of 416 differentially expressed genes in goose feather follicles, among which 248 and 167 were up- and down-regulated, respectively. [file 12864_2020_6562_MOESM3_ESM.docx]

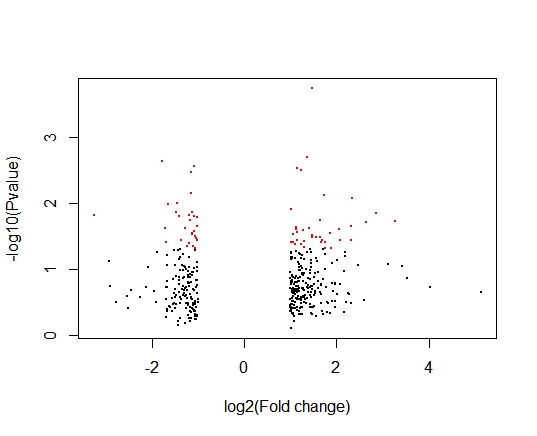


**Figure S3. The Volcanic Plot of differentially expressed genes.** A total of 416 differentially expressed genes in goose feather follicles, among which 248 and 167 were up- and down-regulated, respectively.
