# Supplementary material for: A 14-bp insertion in endothelin receptor B-like (EDNRB2) is associated with white plumage in Chinese geese
Source: BMC Genomics. 2020 Feb 17;21:162. doi: 10.1186/s12864-020-6562-8 (PMC7027040; doi:10.1186/s12864-020-6562-8)
Supplement: Supplementary file 5 — Additional file 5: Figure S5. Complete sequence of grey goose CDS. A pair of primers was used to amplify the complete CDS sequence of gray goose, totaling 1359 bp. [file 12864_2020_6562_MOESM5_ESM.docx]

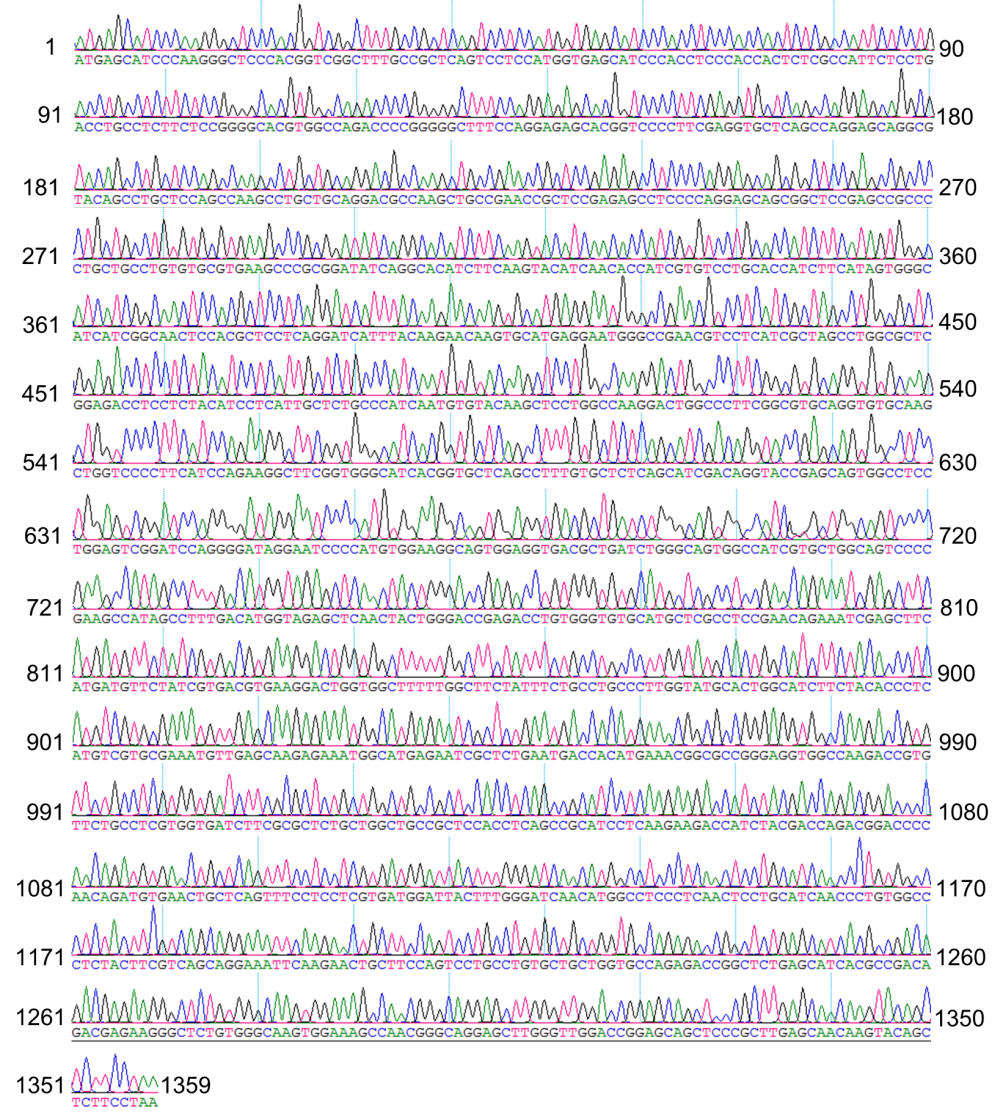


**Figure S5. Complete sequence of grey goose CDS.** A pair of primers was used to amplify the complete CDS sequence of gray goose, totaling 1359 bp.
