# Supplementary material for: A 14-bp insertion in endothelin receptor B-like (EDNRB2) is associated with white plumage in Chinese geese
Source: BMC Genomics. 2020 Feb 17;21:162. doi: 10.1186/s12864-020-6562-8 (PMC7027040; doi:10.1186/s12864-020-6562-8)
Supplement: Supplementary file 6 — Additional file 6: Figure S6. The predicted amino acid sequence of gray goose (left) and after insertion (right). The complete CDS of gray Gang goose (left) was amplified. The amino acid sequences of gray and white goose were predicted. In the gray goose, 452 amino acids were completely translated. However, the insertion (marked in green) may cause a frameshift mutation in the white goose. Pre-termination codon (marked in red) appears at the 664th to 666th nucleotides in the coding region. [file 12864_2020_6562_MOESM6_ESM.docx]

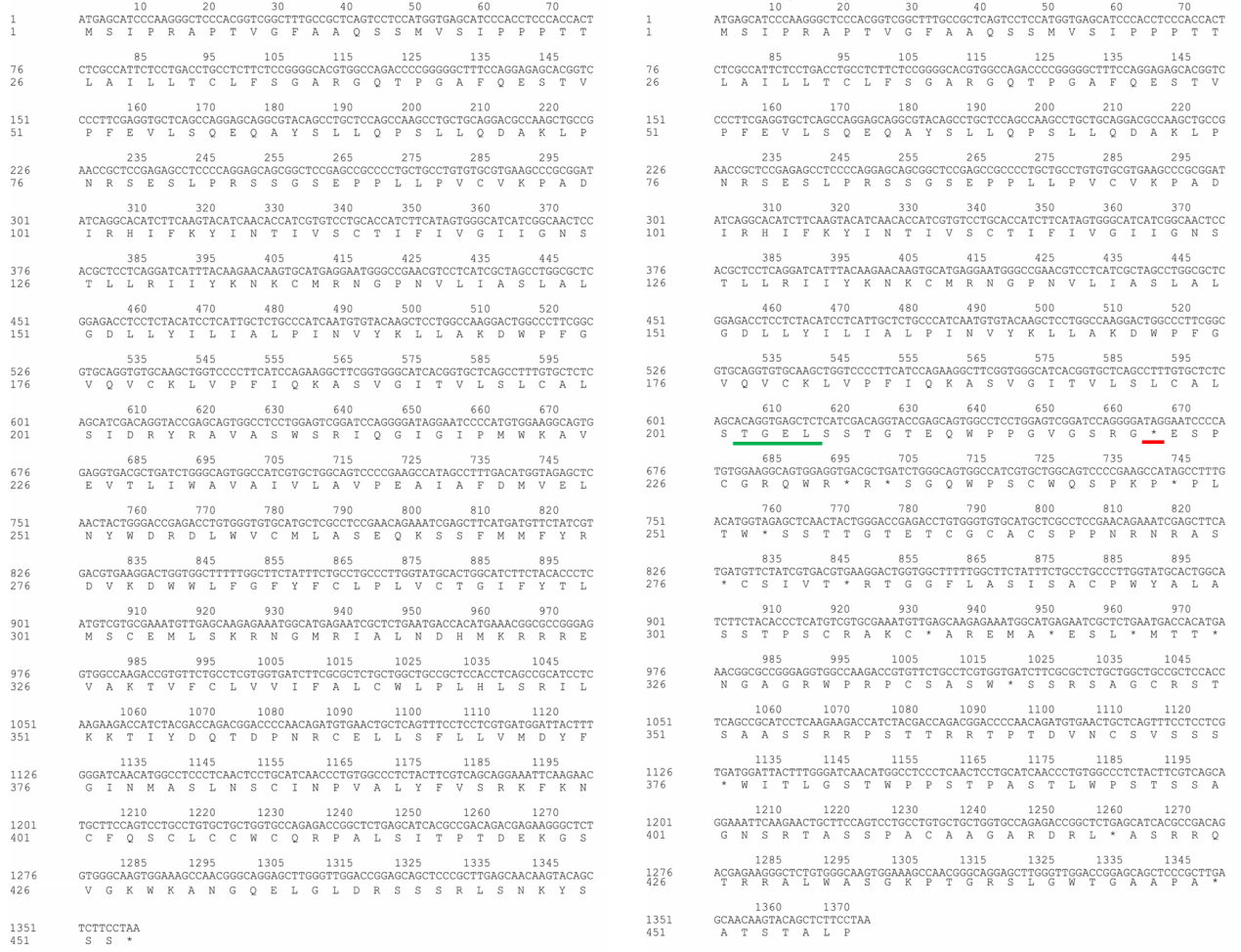


**Figure S6. The predicted amino acid sequence of gray goose (left) and after insertion (right).** The complete CDS of gray Gang goose (left) was amplified. The amino acid sequences of gray and white goose were predicted. In the gray goose, 452 amino acids were completely translated. However, the insertion (marked in green) may cause a frameshift mutation in the white goose. Pre-termination codon (marked in red) appears at the 664th to 666th nucleotides in the coding region.
