# Supplementary material for: A 14-bp insertion in endothelin receptor B-like (EDNRB2) is associated with white plumage in Chinese geese
Source: BMC Genomics. 2020 Feb 17;21:162. doi: 10.1186/s12864-020-6562-8 (PMC7027040; doi:10.1186/s12864-020-6562-8)
Supplement: Supplementary file 7 — Additional file 7: Figure S7. The phenotypic difference between the homozygote (wt/wt) and heterozygote (+/wt) in gray Gang geese. Figure A and C were the homozygote individuals while B and D were heterozygote. The red dotted circles represent the body areas with no pigment distribution. A. The dorsum of homozygote gray goose. B. The dorsum of heterozygote gray goose. C. The primaries of homozygote gray goose. D. The primaries of heterozygote gray goose. [file 12864_2020_6562_MOESM7_ESM.docx]

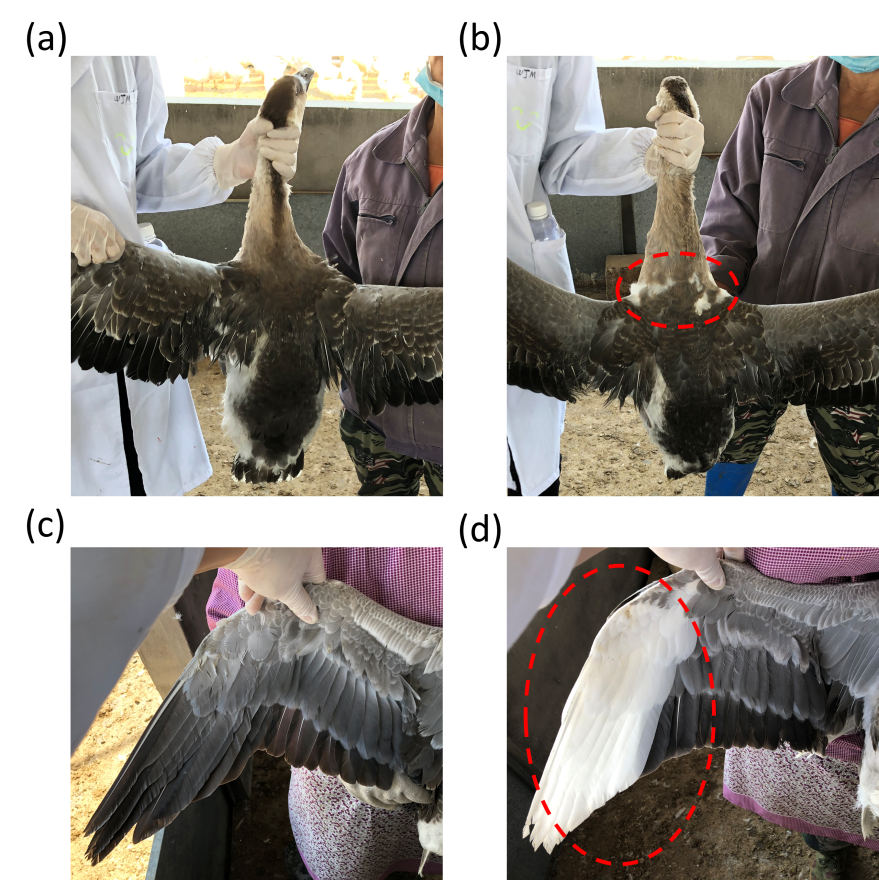


**Figure S7. The phenotypic difference between the homozygote (wt/wt) and heterozygote (+/wt) in gray Gang geese.** Figure A and C were the homozygote individuals while B and D were heterozygote. The red dotted circles represent the body areas with no pigment distribution. **A.** The dorsum of homozygote gray goose. **B.** The dorsum of heterozygote gray goose. **C.** The primaries of homozygote gray goose. **D.** The primaries of heterozygote gray goose.
